# What evidence exists relating the impact of different grassland management practices to soil carbon in livestock systems? A systematic map protocol

**DOI:** 10.1186/s13750-024-00345-2

**Published:** 2024-08-24

**Authors:** Camille Rousset, Carmen Segura, Anina Gilgen, Marta Alfaro, Luís André Mendes, Mike Dodd, Batnyambuu Dashpurev, Mike Bastidas, Julian Rivera, Lutz Merbold, Eduardo Vázquez

**Affiliations:** 1 Integrative Agroecology Group, Research Division Agroecology & Environment, Reckenholzstr. 191, 8046 AgroscopeZurich, Switzerland; 2 Net Zero and Resilient Farming. Rothamsted Research - North Wyke. Okehampton, Okehampton, UK; 3 AgResearch Ltd, Ruakura Research Centre, 10 Bisley Road, Hamilton, 3214 New Zealand; 4 Departamento de Producción Agraria, ETSIAAB, Universidad Politécnica de Madrid (UPM), Madrid, Spain; 5 AgResearch Ltd, Grasslands Research Centre, Private Bag 11008, Palmerston North, 4442 New Zealand; 6 Institute for Meteorology and Climate Research, Atmospheric Environmental Research (IMK-IFU), Karlsruhe Institute of Technology, Garmisch-Partenkirchen, Germany; 7 International Center for Tropical Agriculture (CIAT), Km 17 Recta Cali-Palmira, Palmira, Colombia; 8 Centre for Research On Sustainable Agriculture (CIPAV), Cali, Colombia; 9 Department of Soil Science and Agricultural Chemistry, University of Granada, Granada, Spain

**Keywords:** Pastures, Meadow, Grassland practices, PICO, C content, Climate change mitigation

## Abstract

**Background:**

Grasslands are essential for providing vital resources in the livestock sector and delivering invaluable ecosystem services such as biodiversity and soil carbon (C) sequestration. Despite their critical importance, these ecosystems face escalating threats from human disturbances, human degradation, and climate change, compromising their ability to effectively stock C. Restoring degraded grasslands emerges as a pragmatic and cost-effective approach to tackling climate change. However, the successful implementation of grassland management toward this goal, faces significant challenges. A systematic mapping approach will help to compile a comprehensive global inventory of studies investigating the impact of differing grassland management practices on soil carbon. In addition, the potential for trade-offs with other greenhouse gas emissions further underlines the value of a systematic assessment. This approach aims to identify knowledge clusters (i.e., well-represented subtopics that are amenable to full synthesis) for potential systematic reviews and pinpoint knowledge gaps requiring further primary research efforts, all contributing to a better understanding of the evidence surrounding this topic.

**Methods:**

Following systematic evidence synthesis standards, we developed the question to address in the systematic map protocol using the PICO framework. We established a preliminary search string by combining search terms for the Population (Grasslands), Intervention (management) and Outcome (soil carbon) categories, as well as with one additional group (Study types—to focus on farm and field experiments). We will conduct a comprehensive literature search of relevant peer-reviewed and grey literature using Web of Science, Scopus, CABI platforms, Google Scholar, and specialised websites (e.g., Agrotrop). Searches will be conducted in the English, Spanish, Portuguese, French, German, and Mongolian languages, as per the linguistic capabilities of the research team. The comprehensiveness of the search will be assessed by comparing the literature collected to a test-list of forty relevant articles. The repeatability of the literature screening process will be ensured by a list of inclusion/exclusion criteria and inter-reviewer consistency statistical tests. Data extraction will be organised into four complementary sections (article information, PICO categories, study characteristics, measurable parameters), on which we will perform queries to produce the tables, figures and evidence maps that will compose the systematic map. The results will identify and describe knowledge gaps and clusters.

**Supplementary Information:**

The online version contains supplementary material available at 10.1186/s13750-024-00345-2.

## Background

Globally, grasslands cover about 40% of the Earth’s ice-free surface, and their importance for human livelihood is primarily related to fodder and forage production for the livestock sector [1–3]. Although livestock production is viewed with increasingly criticism given its greenhouse gas (GHG) emissions, grasslands also contribute to global food and nutrition security [4], rural livelihood, and supply valuable ecosystem services across a wide range of geological and climatic conditions [5, 6]. Grasslands are known to help maintain above and belowground biodiversity, filter water and combat erosion [5, 6]. Given that up to 30% of the Earth’s terrestrial C is stored in grasslands [7], they play a key role in climate regulation; therefore, grassland management strategies have received increasing national and global interest as potential pathways for sequestering C [8–11].

Soil C stocks (referring to the mass of carbon in a sample of known bulk density for a nominated depth and commonly restricted to the fraction < 2 mm in size [12]) under grasslands are very vulnerable to disturbance and degradation from human management [13, 14]. These ecosystems can become sinks or sources of CO_2_, depending on multiple variables [13, 15, 16]. Soil organic carbon (SOC) exhibits temporal and spatial variability, being constantly accumulated, decomposed, and mineralized. Although both SOC and soil inorganic carbon (SIC) are present in soils, this paper emphasizes SOC, as it is the primary focus in both scientific and political discussions. Over the past decades, the total area of grassland has decreased while that of arable land has increased, suggesting ongoing conversion of grasslands to croplands [17]. Concurrently, close to 50% of the world's natural grassland has experienced various degrees of degradation [18]. Meanwhile, the demand for livestock products, continues to increase, especially in emerging-economy countries [19]. Less grazing area for greater forage demand has led to an increase in grazing intensity [1, 16], ensuing that a noticeable fraction of the world’s grasslands is hosting a livestock population that exceeds local carrying capacity [20]. This overgrazing as well as other unsustainable grassland management practices (e.g., excessive fertilisation of meadows or biomass removal), can have serious negative impacts for the environment, including negative effects on soil C storage [1, 10, 13, 15]. A better understanding of the effect of different grassland management practices on soil C stocks can help guide efforts to reverse the grassland degradation trend and preserve the important soil C stocks of the world’s grasslands [1, 13, 15, 16].

**Figure 1:**
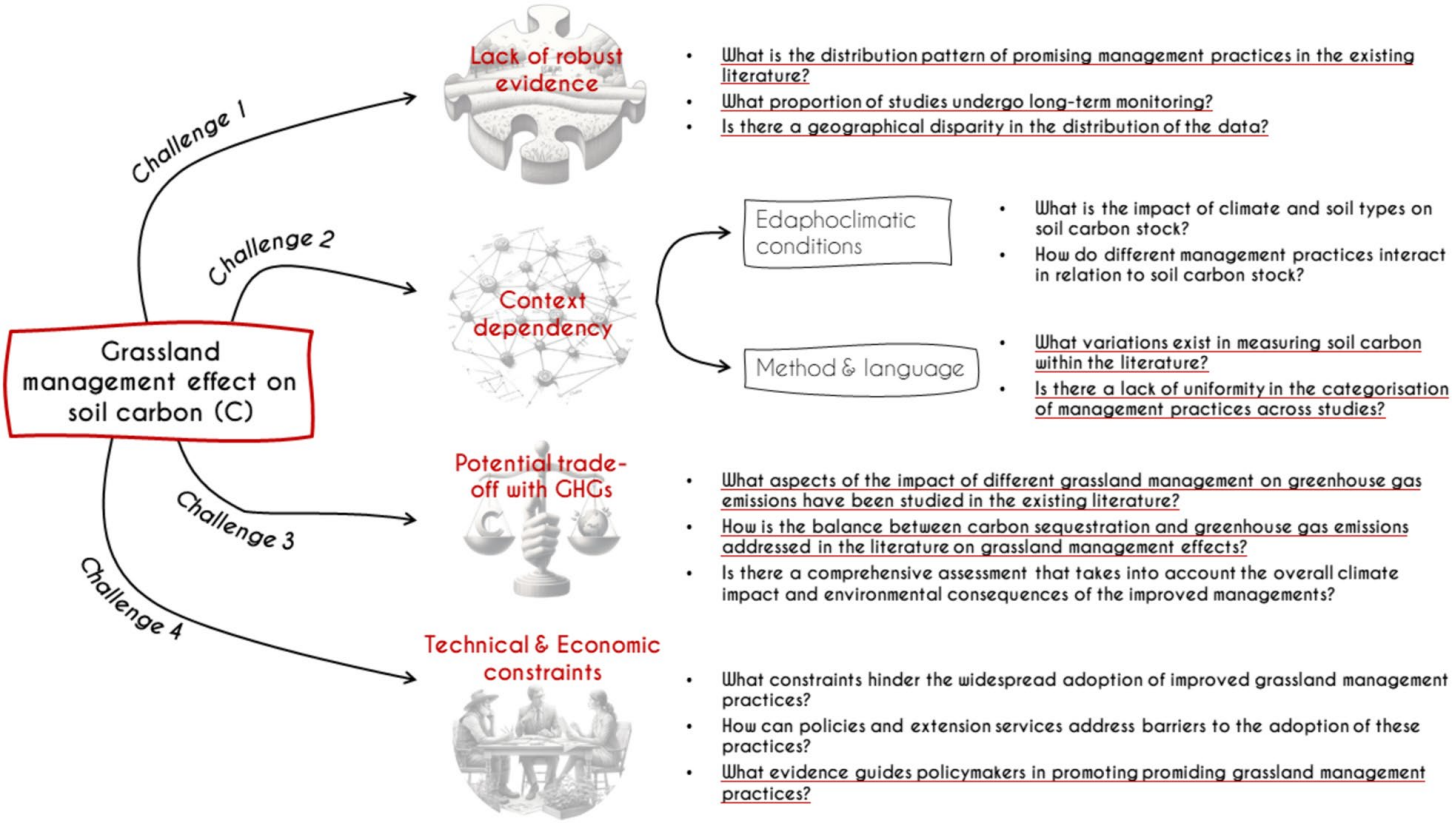
Challenges in implementing grassland management strategies at a global scale: Unveiling critical questions for systematic inquiry. The questions to be addressed in the systematic map are underlined in red.

Considering the large area covered by grasslands globally and that approximately 50% of the area is degraded [14], the potential for C sequestration (referring to the process of capturing and storing atmospheric carbon dioxide in soils) by restoring grasslands offers a very cost-efficient nature-based solution to mitigate climate change [12, 21]. Several reviews and studies have shown the potential for changes in grassland management practices—such as rotational grazing, fertilisation, sowing legumes and improved forages or the establishment of silvopastoral systems—to restore degraded grasslands and increase their soil C stocks [10, 11, 15]. In the context of this study, grassland management refers to a range of human-directed practices implemented with the aim of restoring grasslands while simultaneously enhancing forage quantity and quality, as well as soil C stocks. These may be considered as alternatives to current or locally conventional management practice recognised as leading to degradation. Implementing these strategies on a regional and global scale remains problematic because of four main challenges (Fig. 1): (i) a lack of robust evidence regarding the effect of grassland management practices on soil C stocks, (ii) uncertainties caused by variation in SOC measurements and the complexity of interactions between edaphoclimatic conditions and management practices, (iii) understudied potential trade-offs with other greenhouse gas (GHG) emissions and other environmental consequences resulting from these practice changes, and (iv) the technical and economic constraints faced by farmers, limiting their adoption [10, 13, 22].

Despite a growing number of reviews and articles on the subject, there is still limited evidence that implementing certain promising practices, such as silvopastoral systems or sowing improved forages or legumes, is positive for increasing soil C stocks in grasslands. For example, the meta-analysis on the topic performed by Conant et al. [10] included only one, two and seven studies focused on these practices, respectively. In addition, the geographical distribution of evidence supporting the benefit of most of these practices is not evenly distributed. Africa and Asia are under-represented compared with Europe, North America, South America and Oceania [10]. This is particularly problematic given that Africa and Asia have the highest estimates for potential C storage through grassland restoration [13]. In the context of a research system where modelling studies are increasingly used, the field studies evidence becomes more critical to address substantial uncertainties in the models [8].

The second challenge is the large uncertainty in the predicted soil C sequestration potential of various grassland management practices on a global scale, as their effects are highly context dependent [10, 13, 23]. The C sequestration potential of specific practices will depend on climate, soil characteristics, vegetation (i.e. species composition, presence/absence of C3 or C4 grasses, biological nitrogen fixers etc.), intensity of biomass removal, as well as animal stocking densities and the ingested amount of biomass produced [7, 8, 24–26]. This explains why the study scale is often limited to local or subregional scales [23, 27–30], and thus, upscaling to regional and global scales remains challenging [31]. In addition, the characteristics of management practices are highly site-specific, since what is considered conventional in one situation may be considered alternative in another. Environment and discourse situations can lead to variations in terminology. As an example, the term “silvopastoral systems” encompasses the *dehesa* or *montado* ecosystems of Spain and Portugal, as well as the high-density silvopastoral systems planted with rows of *Leucaena* trees in tropical regions, although their functionality and C sequestration potential are very different [29, 32, 33]. The name used to designate certain practices may also vary from one region or author to another, as there is no scientific standardisation for the name or definition of the practices. This can be the case for rotational grazing strategies which are referred to as “rotational grazing”, “regenerative rotational grazing”, “intensive short-duration rotational grazing”, “cell grazing”, “technograzing”, “mob grazing” or “multi-paddock grazing”, even though the intent of these practices is similar [27, 34–36]. This lack of category uniformity needs to be remedied. It is also essential to note that despite the recent efforts made by international organisations to standardise the methods used to assess changes in soil C stocks [31, 37] there are still significant divergences in measuring, reporting and verifying these changes, which is also a source of uncertainty.

The third challenge is a better understanding of the potential trade-off with other GHG emissions of soil C sequestration and the proposed changes in management practices. While increased soil C stock resulting from changes in grassland management is generally associated with positive environmental outcomes (C sequestration, improved soil health, climate resilience, among others), it is essential to acknowledge potential challenges and negative consequences. For instance, management strategies affecting SOC cycle are likely to affect soil nitrous oxide (N_2_O) emissions as C and N biogeochemical cycles are strongly interlinked [38, 39]. This potential trade-off could offset the climatic benefits of increased soil C stocks. Although many studies and meta-analyses have evaluated the impact of increased soil C on GHG emissions for croplands [38–41], studies evaluating the impact of grassland managements on GHG emissions are rare, with the exception of those evaluating the impact of N fertilisation on N_2_O emissions [42, 43]. A compilation of the evidence could help to identify clusters of knowledge for certain practices and gaps of knowledge that require further empirical research.

The final challenge to the overall implementation of different grassland management practices lies in the technical and economical constraints that may limit adoption by many farmers [10, 13, 22]. The development of policies and extension services to promote those practices as well as to address the adoption barriers are thus necessary [10]. Policymakers and stakeholders usually require a comprehensive body of robust evidence to support the development of policies focused on the promotion of grassland management practices that will achieve restoration goals [44].

Addressing these four challenges (Fig. 1), encourages a detailed collation of the available evidence as well as the development of a comprehensive and systematic database considering the types of management practice being studied, and the contexts in which each study has been carried out. This may help to reduce uncertainties about the potential for soil C enhancement in grasslands in certain regions and potential trade-offs, but also highlight other regions or types of practices where evidence is scarce and further studies are needed. To the best of our knowledge, there is currently no comprehensive, systematic, and updated assessment of the existing evidence on the impact of differing grassland management practices on soil C stocks. The review by Conant et al. [10] is the most comprehensive analysis of existing evidence on the topic to date. However, a sufficient number of articles have been published since this 2017 review, justifying the need for a systematic re-evaluation of the evidence up to the present time. Our objective in this work is not to ascertain the directional impact (positive or negative or neutral) of a practice on soil C. Instead, our focus is on cataloguing the various practices that have been subject to study. Therefore, our approach is a qualitative examination rather than a quantitative assessment of the effects of these practices on SOC.

### Objectives of the review

The impacts of grassland management on soil C have been subject to several conventional literature reviews [11, 13, 15, 16, 45]. To our knowledge, no study has evaluated these impacts systematically. Therefore, the objective of the forthcoming systematic map is to fill this gap of knowledge, answering the following question:

*“What evidence exists relating the impact of different grassland management practices to soil carbon in livestock systems?”*

This systematic map aims to (i) identify a comprehensive list of studies around the globe, on the impact of different grassland management practices on soil C, (ii) supply a robust database of studies with their main descriptive information (meta-data) and to ensure open access availability of this resource, (iii) generate an evidence map indicating the location of the existing studies, (iv) create a list of knowledge clusters [46] of management practices and regions well studied that would be suitable for a full systematic review, and (v) create a list of knowledge gaps of practices and regions underrepresented in studies that warrant further primary research effort. All these outputs will help to understand the existing evidence relating the impact of grassland management practices on soil C.

The PICO (Population, Intervention, Comparison, Outcome) method, a structured approach used in literature reviews to formulate clear research questions, will be used with the following key elements:

#### Population

Managed permanent grasslands and pastures (including silvo-pastoral systems) distributed worldwide. Unmanaged grasslands not subject to animal husbandry are not included.

#### Intervention

Any grassland management practices used as alternative to a locally standard/conventional/nominal/common practice adopted in each study for the purpose of restoring grasslands while maintaining or improving forage quantity and quality and/or soil C stocks.

#### Comparator

The standard/nominal/common local management practices used for comparison in each study.

#### Outcome

Soil C stocks, concentration and related terms (Table 1 and Table S3). While not every article specifically addresses soil C stocks, many studies measure related measurements such as SOC concentration or total C. Similarly, some studies refer to soil health or soil quality, which include different soil properties such as carbon storage. Consequently, we have opted for a broader terminology in our search string to comprehensively cover all aspects of C change related to management practices documented in the literature.

**Table 1:** Substrings relating to the ‘‘Population’’, ‘‘Intervention’’ and ‘‘Outcome’’ elements of the research question and used to construct the general search string for the bibliographic databases

N^**o**^	PICO element	Sub-string
1	Population	Grass* OR prairie* OR meadow* OR forag* OR savanna* OR steppe* OR pastur* OR silvopast* OR pastoral OR rangeland* OR heathland* OR pampa* OR veldt* OR silvo-past* (Forag* was mentioned here as to represent the “forage land” or “forage crops” or any related variations population)
2	Intervention	Improve* OR graz* OR managed OR management OR mow* OR cut* OR fertiliz* OR fertilis* OR "Rest period'' OR irrigat* OR seeding OR legume OR diversification OR lim* OR Manure OR organic OR silvopast* OR silvo-past* OR livestock OR exclusion OR regenerat* OR technogra* OR rotational OR drainage OR burn* OR "fire suppress*"OR restoration OR conservation
3	Outcome	carbon Near/5soil^#^ OR ‘‘organic carbon’’ OR “mineral carbon” "soil organic matter" OR ‘‘carbon sequest*’’ OR ‘‘carbon pool*’’ OR ‘‘carbon sink’’ OR ‘‘ carbon content’’ OR ‘‘soil carbon cycle’’ OR carbon OR ‘‘soil quality’’ OR ‘‘soil stock” OR "soil health” OR SOC OR SOM OR ‘‘Total C’’ OR OC OR OM OR humus

The comparator category is omitted as it is inherently subsumed within the population and intervention string

* Is a wildcard returning several variations of the search term

^#^ Only available in Web of Science, replace with “soil carbon” for other bibliographies

## Methods

This systematic map has been designed based on the Collaboration for Environmental Evidence Guidelines and Standards for Evidence Synthesis in Environmental Management [47]. The preparation of this systematic map protocol has been made following the ROSES reporting standards [48].

### Searching for articles

While the primary focus will be on peer-reviewed articles, other forms of literature such as book chapters or conference proceedings will also be considered. The year of publication will not be restricted.

#### Academic bibliographic databases

The academic bibliographic databases used for this study are listed in Table 2. The literature search will be mainly done using the subscriptions of the Swiss Consortium. The search string was built up in agreement by all authors of the study. The search string was designed to include three substrings representing the *population*, *intervention* and *outcome* of the PICO question and connected by the Boolean operator “AND” (Table 1). The search strings will be adapted to the syntax requirements of each database (Table 2). The searches in bibliography databases will be performed in English using the refinement criterion ‘Language’ in the academic databases. Additionally, an initial screening process will be performed on the academic bibliographic platforms to eliminate articles that are clearly irrelevant based on their subject categories. For example, certain articles may have been categorised under art, sociology, or veterinary topics subsequent to the primary search.

**Table 2:** Academic bibliographic databases and their respective search strings and locations.

Database name	Search string	Search within
Web of sciences core collections	Grass* OR prairie* OR meadow* OR forag* OR savanna* OR steppe* OR pastur* OR silvopast* OR pastoral OR rangeland* OR heathland* OR pampa* OR veldt* OR silvo-past* AND Improve* OR graz* OR managed OR management OR mow* OR cut* OR fertiliz* OR fertilis* OR ‘‘Rest period’’ OR irrigat* OR seeding OR legume OR diversification OR lim* OR Manure OR organic OR silvopast* OR silvo-past* OR livestock OR exclusion OR regenerat* OR technogra* OR ‘‘rotational’’ OR drainage OR burn* OR ‘‘fire suppress*’’OR restoration OR conservation AND carbon Near/5 soil^#^ OR ‘‘organic carbon’’ OR “mineral carbon” ‘‘soil organic matter’’ OR ‘‘carbon sequest*’’ OR ‘‘carbon pool*’’ OR ‘‘carbon sink’’ OR ‘‘ carbon content’’ OR ‘‘soil carbon cycle’’ OR carbon OR ‘‘soil quality’’ OR ‘‘soil stock” OR ‘‘soil health” OR SOC OR SOM OR ‘‘Total C’’ OR OC OR OM OR humus	Topic (Searches title, abstract and author keywords)
Scopus		Article title, abstract, keywords
CABI digital library		Abstract
ProQuest natural science collection		Everywhere but not full text—NOFT
Directory of open access journals (DOAJ)	Full sentence: an ‘‘Improved management’’ effect on soil organic carbon (e.g. Grazing effect on soil organic carbon)	Abstract
Agrotrop	Full sentence: an ‘‘improved management’’ effect on soil organic carbon (e.g. Grazing effect on soil organic carbon)	All

* is a wildcard returning several variations of the search term

^#^ only available in Web of Sciences core collections, replace with “soil carbon” for other bibliographies

To ensure that the systematic map remains up-to-date, several strategies for updating searches will be implemented. First, regular searches will be conducted in relevant databases and repositories to identify newly published literature related to the impact of grassland management practices on soil C. These searches will be conducted at predefined intervals throughout the systematic mapping process. Additionally, automated alerts will be set up in databases and search engines to notify the research team of any newly published articles that meet the inclusion criteria. These alerts will be tailored to specific search terms and filters used in the systematic map protocol.

#### Grey literature

The search for grey literature will be made using the Google Scholar engine which has been demonstrated to be a useful tool for identifying grey literature [49]. We will evaluate grey literature in five languages in addition to English, corresponding to the native languages of all contributing authors of this article. This approach aims to include studies that may have been overlooked during the English-language academic database search. Therefore, simplified search strings in English, Spanish, French, German, Mongolian and Portuguese will be used (Table S1). In the case of a very large number of matches, the analysis will be limited to the first 500 hits. We acknowledge the limitation of using only Google Scholar for this grey literature search, as it may not capture all types of grey literature for the different languages tested.

#### Supplementary searches

The protocol preparation team may contact other relevant researchers and stakeholders to ask for additional literature related to the topic. Furthermore, the bibliographies of some selected papers (particularly reviews and meta-studies) relevant on the topic will be screened aiming to find relevant bibliographies (e.g. snowballing/backward citation searching). Theses and dissertation on the topic will be examined.

#### Estimating the comprehensiveness of the search

To assess the comprehensiveness of the search, we prepared a list of reference articles that were considered relevant for this systematic map based on the experience of the authors of this article (Table S2). We have checked whether the reference articles were captured by our search strategy both in academic bibliographic databases used and in Google Scholar. Using the mentioned search strings in Table 1 in a preliminary search, articles have been found in both the academic bibliographic databases as well as in Google Scholar, confirming the comprehensiveness of our search.

### Article screening, study eligibility criteria and analysis

Both the article screening process and data extraction will be conducted using the Covidence software﻿ [50]. Covidence is a web-based collaboration software platform that streamlines the production of systematic and other literature reviews.

#### Screening process

Since we anticipate that various search databases may duplicate references, we will first eliminate duplicates using the Covidence software tool which identifies duplicates on the basis of the DOI or title. We will also classify the articles by authors to facilitate the identification of any duplicates that may have been overlooked by the software. Then, the studies selected will be screened to decide their inclusion within the systematic map based on the eligibility criteria described below. The screening will be conducted firstly using to the titles, keywords and abstract of the studies, and secondly, using the full-text. To ensure repeatability of the selection process during the title and abstract screening phases, a subsample of 200 articles will be randomly selected and independently reviewed and tested for consistency of selection by all members of the selection team. If applicable, disagreements in the eligibility criteria will be discussed and clarified to ensure the accurate repeatability of the screening process. This repeatability procedure will be reiterate with another random subset of articles until a repeatability of 95% among all the members is reached. Even when consistency will be sufficient (> 95%), reviewers will discuss and solve the remaining disagreements to ensure a high replicability. Special attention will be given to avoid situations where screening team members, who are authors of articles, review their own work. A similar methodology will be applied during the full-text screening phase, using a subsample adjusted to correspond to 5% of the number of articles validated during the title and abstract screening.

#### Eligibility criteria

We will screen the studies regarding the population, intervention, comparator, outcome, and study design listed in Table S3.

##### Eligible population

To be included in the systematic map, studies must evaluate permanent grasslands managed for livestock production (including those that have a woody vegetation component, such as silvopastoral systems). This excludes unmanaged grasslands without human intervention and grasslands managed for biofuel production or recreation. Managed natural grasslands with naturally occurring indigenous grass communities will be included. Grassland-cropping rotations or so called “temporary grasslands” [51] will be excluded, since the objective of the addition of a grassland phase within the crop rotation schemes is a means to mitigate the environmental impacts of intensive arable cropping systems. Likewise, forests including livestock grazing with the objective of benefitting the forest management as described by Öllerer et al. [52], will be excluded as the targeted grazing is a means for enhancing forest management rather than an end. Due to the unique carbon dynamics observed in wetlands and peatlands (or mires) in contrast to other grassland ecosystems, they will be excluded from our study [53]. Grazing systems dominated by woody species will also be omitted from the study (i.e. shrublands, scrublands).

##### Eligible intervention

As mentioned above, to be included in the systematic map, studies must evaluate any permanent grassland management practices used as alternatives to a locally standard/conventional/nominal/common practice adopted in the studies. These practices should aim to restore grasslands while maintaining or improving forage quantity and/or quality and/or soil C stocks. Whether or not the objective is achieved at the end of the experiment, will have no impact on the selection. This definition combines a variety of practices as the used permanent grassland management practice depends strongly on the context of application. Grassland management practices used with objectives other than livestock production or improving soil health, such as the reclamation of polluted soils or biomass production for energy, will not be included. It is important to emphasise that the management practise should be undertaken with the objective of maintaining or increasing grassland quality and yield, rather than facilitating supplementary planting. In the latter case, the grassland is considered as a service plant, and will not be included in the study. The implementation of grazing exclusion, whether through enhanced management practices such as meadow establishment or through temporary exclusion measures such as rest periods, will be included. However, studies focusing on abandoned grasslands will be excluded.

##### Eligible comparator

An eligible comparator includes any locally standard/conventional/nominal/common practice grassland used by the authors in the study as a comparator to a specific grassland management practice. We will only consider as a comparator those practices which investigate grasslands, i.e. other comparators such as arable cropping agriculture, forest, abandoned grasslands, park or urban soils will be excluded. In addition, we will consider as a comparator, those grassland management practices performed with a different intensity than the targeted intervention practice. If a study involves a comparison between a grassland system and a crop system (or forest or native ecosystems), along with a comparison to a silvopastoral system or another grassland management, it will be included based on the latter comparison.

##### Eligible outcome

To be included in the systematic map, the study must report either the soil C stock, C concentration or any other terms mentioned in Table S3, in their abstract, keywords or title. In soil science, agricultural and environmental studies, providing soil C stock, C concentration, or related metrics is common as a description of the soil of study site even when soil C is not the primary outcome objective [54–56]; however, for this study, we included only studies where soil C is one of the primary focuses, and should therefore be indicated in the abstract, keywords, or title, to keep the review manageable considering our available resources.

##### Eligible study design and other characteristics

We will include field studies under realistic conditions that provide experimental data. Mesocosm experiments (e.g., lab incubations or pot experiments) will be excluded unless they are part of a combined field study. In this study, modelling-only articles will be excluded from the selected list of publications. Models rely on various data inputs, and the quality and reliability of these inputs can vary widely among studies [57]. This variability can introduce uncertainty and affect the overall reliability of the systematic map's conclusions.

We will include both the control-impact studies (intervention *vs* comparator) and the before-after intervention studies (comparing soil carbon over time) if a control is provided to evaluate the change in the soil C. We will accept both studies performed on experimental plots as well as the comparison of real farms. We will not restrict the number of replicates and the study duration. In cases where two or more studies report the same data from the same experiment, we will choose one of the studies and exclude others, except if they provide complementary supporting environmental or management information about the site.

### Study validity assessment

An assessment of study validity (in terms of the methods, assumptions or peer-review processes used) will not be performed because the objective of the systematic map is to compile all the existing evidence rather than to assess the quality or validity of the existing data [47]. However, during the extraction phase, data on the quality of the studies (e.g., study characteristics outlined in Table 3) will be gathered and subsequently reported in the systematic map. We will remain open to the possibility of incorporating a validity assessment, especially if deemed beneficial based on the quantity and nature of the studies included.

**Table 3:** List of variables to be extracted from the relevant studies

Sections	Variables names	Categories	Information	Example
Article information	Title		Full title of the article	Regenerative rotational grazing management of dairy sheep increases springtime grass production and topsoil carbon storage
	Publication date		Year	2021
	First author last name			Díaz de Otárola
	Journal		Full name	Ecological Indicators
	Doi			https://doi.org/10.1016/j.ecolind.2021.107484
	Language		Language of the main text	English
PICO categories	Population (Type of grasslands)	Grazed pasture	Experimental refer to experimental grass plots that are not harvested/grazed	Grazed pasture
		Meadow		
		Experimental		
	Intervention	Grazing management	A short description for each intervention will be given	Grazing management
		Fertilisation management		
		Silvopasture		
		Mowing/cutting management		
		Liming		
		Irrigation		
		Drainage		
		Burning		
		Vegetation management		
		Other		
	Intervention details		Full name	Regenerative rotational grazing
	Comparator	Grazing	A short description for each comparator will be given	Grazing
		Fertilisation		
		Silvopasture		
		Mowing/Cutting		
		Liming		
		Irrigation		
		Vegetation		
		Natural grassland		
		Other		
	Comparator details		Full name	Conventional rotational grazing
	Outcome	Soil C stocks	Outcome of C values	Soil C stocks
		Soil OM stocks		
		Soil C concentration		
		Soil total C		
		Soil OM concentration		
		Other (to specify)		
Study characteristics	Country of the study		Can be multiple (put a ; to separate)	Spain
	Coordinates		Information to include if available. Indicate NA if not available	42º51ʹ11.41′′ N, 2º37ʹ27.20′′ W
	Altitude (m)		Information to include if available. Indicate NA if not available	567
	MAP (mm)		Information to include if available. Indicate NA if not available	855
	MAT (°C)		Information to include if available. Indicate NA if not available	12
	Study type	Experimental field plot	Lab and mesocosms studies will be rejected, except if they are combined to field exepriment.	Experimental field plot
		Real farms		
		Combined study (experimental/field)		
	Years since establishment		How long has the intervention (improved management practices) been in place?	6
	Study period (month)	value/single	The period taken into account for the study or a single sample.	single
	Paired study	Yes		YES
		No		
	Number of replicates			3
	Replication type	Spatially independent replicats		Spatially independant replicats
		Pseudoreplication		
		Other		
	Number of depth increments			1
	Max. soil depth (cm)			10
Measurable parameters	Bulk density (mean value in g/cm^3^)		Mean value across treatments. Indicate NA if not available	NA
	Soil pH (mean)		Mean values across treatments. Indicate NA if not available	NA
	Soil texture		Indicate NA if not available	Clay loam
	Clay content (%)		Mean values across treatments. Indicate NA if not available	31.9
	N_2_O fluxes	Yes		No
		No		
	CO_2_ fluxes	Yes		No
		No		
	CH_4_ fluxes	Yes		No
		No		
	Most common plant species	Grasses	Only take into account the main categories (herbs, legumes, forbes) or the characteristics of their metabolism (C3, C4 or CAM). Multiple choice possible. In the case where intervention is based on sowing improved forages or sowing legumes, indicate only the improved sown plant.	Grasses
		Legumes		
		Forbs		
		Mixture (incl. Legumes)		
		C3		
		C4		
		CAM		
		Not mentioned		

This list is not exhaustive and subject to change upon the reviewing process

### Data coding and extraction strategy

The meta-data will be extracted, organised and compiled in Excel files for all the relevant selected studies. The data extracted from the articles will be organised into four complementary sections (article information, PICO categories, study characteristics, measurable parameters, Table 3), on which we will perform queries to produce the tables, figures and evidence maps that will compose the systematic map. Some variables will be represented as multiple-choice options. The extraction, organisation and compilation will be performed by the co-authors in a subset of the relevant studies included (15 articles), to check the consistency between the co-authors in this process. We will report in the systematic map the repeatability of each of the extracted variables.

### Study mapping, presentation and analysis

In the systematic map we will provide answers to the primary question developed in the present protocol and the secondary questions presented in Fig. 1, in the form of descriptive texts supported by tables, figures and maps. As the table described in the data organisation strategy (Table 3) might be further developed during full-text assessment, we will provide all final table structures and contents in the upcoming publication, as well as the final database produced in the frame of this review. Descriptive statistics in R or other open-source software will be used to analyse the results. Among the results, we will include “heat maps” that cross-tabulate two variables and illustrate the quantity of evidence (number of studies) within each cell of the table. These heat maps will explore various combinations of variables, such as types of grassland management practices in relation to the study country or study variables (Table 3). Furthermore, an evidence atlas will be generated using study latitude and longitude meta-data and presenting studies on an interactive cartographic map. Detailed information about the articles included/excluded at the different stages of the screening process will be reported, in addition to any eventual modifications to the present protocol. Finally, the detection of knowledge gaps will involve the identification of subtopics that are either unexplored or underrepresented in the literature. For that objective, we will use heat maps or bar plots to highlight these gaps. Subtopics devoid of studies or a small number of studies will be documented. Similarly, the evidence clusters will be identified using heat maps and bar plots, and subtopics with more evidence available will be highlighted.

## Supplementary Information

Additional file 1. ROSES checklist for the systematic map.

## Data Availability

Data sharing is not applicable to this protocol article as no datasets were generated or analysed during the current study.
