# Supplementary material for: What evidence exists relating the impact of different grassland management practices to soil carbon in livestock systems? A systematic map protocol
Source: Environ Evid. 2024 Aug 24;13:22. doi: 10.1186/s13750-024-00345-2 (PMC11344701; doi:10.1186/s13750-024-00345-2)
Supplement: Supplementary file 1 — Additional file 1. ROSES checklist for the systematic map. [file 13750_2024_345_MOESM1_ESM.docx]

Supplementary Tables

**Table S1 The simplified search string used in the google scholar engine to identify grey literature and its translation into Spanish, French, Portuguese, Mongolian and German.**

| **Language** | **Search string** |
| --- | --- |
| English | Grassland management effect on soil organic carbon |
| Spanish | Efecto del manejo de los pastos en el carbono orgánico del suelo |
| French | Effet de la gestion des prairies sur le carbone organique du sol |
| Portuguese | Efeito da gestão das pastagens no carbono orgânico do solo |
| Mongolian | Хөрсний органик нүүрстөрөгчийн хуримтлалд бэлчээрийн менежментийн нөлөө |
| German | Auswirkungen der Grünlandbewirtschaftung auf den organischen Kohlenstoff im Boden |

**Table S2 A list of 40 relevant articles used as a reference to validate the search strategy.**

| **N^o^** | **First author** | **Date** | **Title** | **doi** |
| --- | --- | --- | --- | --- |
| 1 | S. Eze | 2018 | Soil organic carbon stock in grasslands: Effects of inorganic fertilizers, liming and grazing in different climate settings | https://doi.org/10.1016/j.jenvman.2018.06.013 |
| 2 | R.T. Conant | 2017 | Grassland management impacts on soil carbon stocks: a new synthesis | https://doi.org/10.1002/eap.1473 |
| 3 | D. Herfurth | 2015 | How does soil particulate organic carbon respond to grazing intensity in permanent grasslands? | <https://doi.org/10.1007/s11104-015-2528-z> |
| 4 | M.J. Pringle | 2014 | The effect of pasture utilization rate on stocks of soil organic carbon and total nitrogen in a semi-arid tropical grassland | https://doi-org/10.1016/j.agee.2014.05.013 |
| 5 | C. Poeplau | 2021 | Grassland soil organic carbon stocks along management intensity and warming gradients | https://doi.org/10.1111/gfs.12537 |
| 6 | M.E. Mcsherry | 2013 | Effects of grazing on grassland soil carbon: a global review | https://doi-org/10.1111/gcb.12144 |
| 7 | N. He | 2012 | Land-use impact on soil carbon and nitrogen sequestration in typical steppe ecosystems, Inner Mongolia | https://doi-org/10.1007/s11442-012-0968-4 |
| 8 | T. Zhan | 2023 | Differential effects of grazing intensity on carbon sequestration in arid versus humid grasslands across China | https://doi.org/10.1016/j.scitotenv.2023.163221 |
| 9 | A. Chabbi | 2022 | Managing grasslands to optimize soil carbon sequestration | http://dx.doi.org/10.19103/AS.2022.0106.17 |
| 10 | C.E Riggs | 2015 | Nitrogen addition changes grassland soil organic matter decomposition | https://doi.org/10.1007/s10533-015-0123-2 |
| 11 | X. Díaz de Otárola | 2021 | Regenerative rotational grazing management of dairy sheep increases springtime grass production and topsoil carbon storage | <https://doi.org/10.1016/j.ecolind.2021.107484> |
| 12 | R.C. Byrnes | 2018 | A global meta-analysis of grazing impacts on soil health indicators | <https://doi.org/10.2134/jeq2017.08.0313> |
| 13 | S.K. Jones | 2006 | Carbon sequestration in a temperate grassland; management and climatic controls | <https://doi.org/10.1111/j.1475-2743.2006.00036.x> |
| 14 | Y. Bai | 2022 | Grassland soil carbon sequestration: Current understanding, challenges, and solutions | https://doi.org/10.1126/science.abo2380 |
| 15 | A. Gilmullina | 2020 | Management of grasslands by mowing versus grazing–impacts on soil organic matter quality and microbial functioning | https://doi.org/10.1016/j.apsoil.2020.103701 |
| 16 | K. Phukubye | 2022 | On the impact of grassland management on soil carbon stocks: a worldwide meta-analysis | <https://doi.org/10.1016/j.geodrs.2021.e00479> |
| 17 | L. L. de Souza Almeida | 2021 | Soil carbon and nitrogen stocks and the quality of soil organic matter under silvopastoral systems in the Brazilian Cerrado | <https://doi.org/10.1016/j.still.2020.104785> |
| 18 | J. Ca | 2022 | C and N stocks in silvopastoral systems with high and low tree diversity: Evidence from a twenty-two year old field study | <https://doi.org/10.1016/j.scitotenv.2022.155298> |
| 19 | J. C. Suárez | 2024 | Agroforestry systems affect soil organic carbon stocks and fractions in deforested landscapes of Amazonia | <https://doi.org/10.1007/s10457-023-00949-6> |
| 20 | J.B. Adewopo | 2014 | Management intensification impacts on soil and ecosystem carbon stocks in subtropical grasslands | <https://doi.org/10.2136/sssaj2013.12.0523> |
| 21 | R. T. Conant | 2001 | Grassland management and conversion into grassland: effects on soil carbon | [https://doi.org/10.1890/1051-0761(2001)011[0343:GMACIG]2.0.CO;2](https://doi.org/10.1890/1051-0761(2001)011%5b0343:GMACIG%5d2.0.CO;2) |
| 22 | G. B. De Deyn | 2011 | Additional carbon sequestration benefits of grassland diversity restoration | <https://doi.org/10.1111/j.1365-2664.2010.01925.x> |
| 23 | E. M.F. Maia | 2009 | Effect of grassland management on soil carbon sequestration in Rondonia and Mato Grosso states, Brazil | <https://doi.org/10.1016/j.geoderma.2008.11.023> |
| 24 | A. Olaya-Montes | 2020 | Restoring soil carbon and chemical properties through silvopastoral adoption in the Colombian Amazon region | <https://doi.org/10.1002/ldr.3832> |
| 25 | D.C. de Oliveira | 2022 | Changes in soil carbon and soil carbon sequestration potential under different types of pasture management in Brazil | <https://doi.org/10.1007/s10113-022-01945-9> |
| 26 | I. Costa de Freitas | 2020 | Agrosilvopastoral systems and well-managed pastures increase soil carbon stocks in the Brazilian cerrado | <https://doi.org/10.1016/j.rama.2020.08.001> |
| 27 | G. R. Castellano | 2022 | Carbon soil storage and technologies to increase soil carbon stocks in the South American savanna | <https://doi.org/10.3390/su14095571> |
| 28 | S. Meyer | 2012 | Free and protected soil organic carbon dynamics respond differently to abandonment of mountain grassland | https://doi.org/10.5194/bg-9-853-2012 |
| 29 | N. Rogiers | 2005 | Effect of land management on ecosystem carbon fluxes at a subalpine grassland site in the Swiss Alps | https://doi.org/10.1007/s00704-004-0099-7 |
| 30 | M. Zeeman | 2010 | Management and climate impacts on net CO_2_ fluxes and carbon budgets of three grasslands along an elevational gradient in Switzerland | https://doi.org/10.1016/j.agrformet.2010.01.011 |
| 31 | S. Keel | 2019 | Loss of soil organic carbon in Swiss long-term agricultural experiments over a wide range of management practices | <https://doi.org/10.1016/j.agee.2019.106654> |
| 32 | J. Moll-Mielewczik | 2023 | Organic carbon contents of mineral grassland soils in Switzerland over the last 30 years | https://doi.org/10.1016/j.agee.2022.108258 |
| 33 | N. Rogiers | 2008 | Impact of past and present land-management on the C-balance of a grassland in the Swiss Alps | https://doi.org/10.1111/j.1365-2486.2008.01680.x |
| 34 | L. Rodriguez | 2021 | Agroforestry systems in the Colombian Amazon improve the provision of soil ecosystem services | https://doi.org/10.1016/j.apsoil.2021.103933 |
| 35 | N. Teutscherova | 2021 | Intensive short-duration rotational grazing is associated with improved soil quality within one year after establishment in Colombia. | https://doi.org/10.1016/j.apsoil.2020.103835 |
| 36 | J. Chang | 2021 | Climate warming from managed grasslands cancels the cooling effect of carbon sinks in sparsely grazed and natural grasslands | https://doi.org/10.1038/s41467-020-20406-7 |
| 37 | I. Feigenwinter | 2023 | Large inter-annual variation in carbon sink strength of a permanent grassland over 16 years: Impacts of management practices and climate | https://doi.org/10.1016/j.agrformet.2023.109613 |
| 38 | K.M Varsha | 2019 | High density silvopasture systems for quality forage production and carbon sequestration in humid tropics of Southern India | https://doi-org/10.1007/s10457-016-0059-0 |
| 39 | D. Whitehead | 2018 | Management practices to reduce losses or increase soil carbon stocks in temperate grazed grasslands: New Zealand as a case study. | https://doi.org/10.1016/j.agee.2018.06.022 |
| 40 | C. Wade | 2022 | Does Grazing Affect Soil Carbon in Subtropical Humid Seminatural Grasslands? | https://doi-org/10.1016/j.rama.2021.09.004 |

**Table S3 A list of the eligibility criteria for the screening process.**

| Population |  |  |
| --- | --- | --- |
| **Include** |  | **Exclude** |
| Permanent grasslands, natural grasslands (indigenous, naturally occurring grass communities but managed), silvopastures systems; Any grazing animals will be included (cows, pigs, chickens, goats…); Any cutting managements will be included. |  | Rotational grassland (or also called temporary grasslands or ley);  Study focusing only on forests;  Study focusing only on croplands or arablelands; Industrial or biofuel production grassland;  Wetlands and peatlands have distinct carbon dynamics compared to grasslands so they will be excluded; Dominant woody species and shrubs grazing systems. |
|  |  |  |
|  |  |  |
|  |  |  |
|  |  |  |
|  |  |  |
|  |  |  |
|  |  |  |
|  |  |  |
|  |  |  |
| Intervention/Exposure |  |  |
| **Include** |  | **Exclude** |
| Every grassland managements (fertilisation, grazing intensity, liming, irrigation, different varieties...); Grazing exclusion is included (such as meadow). It can also be temporal exclusion (such as rest period); Regenerative ranching; Studies with one or more grassland managements;  Grassland has to be the primary crop; Prescribed burning. |  | Land use change (e.g. studies looking at C evolution following a conversion from maize field to grassland); Abandoned grasslands (no more human intervention, no production); Wild fire;  Grassland as a service crop to enhance another main crop production (e.g. vignards, maiz). |
|  |  |  |
|  |  |  |
|  |  |  |
|  |  |  |
|  |  |  |
|  |  |  |
|  |  |  |
|  |  |  |
|  |  |  |
| Comparator |  |  |
| **Include** |  | **Exclude** |
| Solely grasslands (including silvopastoral systems and natural grasslands);  If a study compares a grassland system with a crop system (or forest or native ecosystems) as well as with a silvopastoral system or another grassland management, it will be included due to the latter. |  | Croplands or Forests or Native ecosystems; Abandoned grassland/unmanaged grassland Invasive species sown. |
|  |  |  |
|  |  |  |
|  |  |  |
|  |  |  |
|  |  |  |
|  |  |  |
|  |  |  |
| Outcome |  |  |
| **Include** |  | **Exclude** |
| "soil carbon" OR "organic carbon" OR “mineral carbon” "soil organic matter" OR "carbon sequestration" OR "carbon pool" OR "carbon sink" OR "soil carbon content" OR "soil carbon cycle" OR carbon OR "soil quality" OR "soil stock” OR "soil health” OR SOC OR SOM OR "Total C" |  | CO2 emissions only |
|  |  |  |
|  |  |  |
|  |  |  |
|  |  |  |
|  |  |  |
|  |  |  |
|  |  |  |
| Study Characteristics |  |  |
| **Include** |  | **Exclude** |
| Experimental field studies, farm studies, combined field/mesocosms study (if mentioned in the abstract); Remote sensing studies. |  | Literature review, opinion, systematic review (but references will be look at); Lab studies (destructive soil samples); Modeling studies. |
|  |  |  |
|  |  |  |
|  |  |  |
|  |  |  |
|  |  |  |
|  |  |  |
|  |  |  |
